# Elevated Creatine Kinase in Mucolipidosis Type IV: Investigating an Atypical Manifestation and Its Clinical Complications

**DOI:** 10.7759/cureus.105879

**Published:** 2026-03-26

**Authors:** Zahra Alsahlawi, Hasan M Isa, Lan Ghassan, Reem A Taha, Yehia Elshafey, Amin Aboultaif, Malak W Sary, Batool H Isa

**Affiliations:** 1 Department of Pediatrics, Arabian Gulf University, Manama, BHR; 2 Department of Pediatrics, Salmaniya Medical Complex, Manama, BHR; 3 Department of Pediatrics, Royal College of Surgeons in Ireland, Busaiteen, BHR

**Keywords:** creatine kinase elevation, lysosomal storage disorder, mcoln1, mucolipidosis type iv, pediatric myopathy

## Abstract

Mucolipidosis type IV (MLIV) is a rare autosomal recessive lysosomal storage disorder caused by pathogenic variants in the *MCOLN1* gene on chromosome 19. It is characterized by neurodevelopmental delay, progressive visual impairment, and gastrointestinal abnormalities. Here, we report a 10-year-old boy from Bahrain with MLIV who presented with global developmental delay, spastic quadriparesis, severe visual impairment, gastrointestinal manifestations, and persistent elevation of creatine kinase (CK). Serial brain MRI demonstrated delayed myelination, corpus callosum thinning, and periventricular leukomalacia. Diagnosis was confirmed by whole-exome sequencing, identifying a homozygous *MCOLN1* variant. Management was supportive, including nutritional optimization, iron supplementation, intermittent blood transfusions, and multidisciplinary follow-up. This case expands the phenotypic spectrum of MLIV by highlighting persistent hyperCKemia, supporting possible secondary myopathic involvement, and emphasizing that elevated CK does not exclude MLIV in non-Ashkenazi populations.

## Introduction

Mucolipidosis type IV (MLIV) is a rare autosomal recessive lysosomal storage disorder caused by pathogenic variants in the *MCOLN1* gene on chromosome 19, which encodes the transient receptor potential (TRP) cation channel, mucolipin-1 [1]. Mucolipin-1 regulates endolysosomal trafficking and membrane fusion; its dysfunction disrupts late endosome-lysosome fusion and impairs lysosomal homeostasis [2].

MLIV is characterized by the accumulation of phospholipids and gangliosides in lysosomes, resulting in severe neurological and ophthalmological deterioration [3]. It primarily affects the nervous system, eyes, and gastrointestinal tract, with secondary effects on other systems. Affected individuals typically present in infancy with severe psychomotor delay, intellectual disability, hypotonia, achlorhydria with hypergastrinemia, and visual impairment due to corneal clouding and progressive retinal dystrophy [4,5].

Diagnosis is typically based on the characteristic clinical phenotype, supportive findings such as hypergastrinemia and lysosomal inclusions on skin biopsy, and confirmatory molecular testing demonstrating pathogenic variants in *MCOLN1 *[1].

Fewer than 120 cases of MLIV have been reported worldwide, with an estimated incidence of approximately one in 40,000 live births [6,7]. MLIV is inherited as an autosomal recessive trait, most commonly reported in individuals of Ashkenazi Jewish descent due to founder mutations in the *MCOLN1 *gene. Cases outside this population are rare and often associated with familial mutations [1].

This case report describes a young Bahraini child with MLIV presenting with elevated creatine kinase (CK) levels, a finding not classically associated with MLIV and suggestive of potential secondary myopathic involvement. Persistent elevation of serum CK levels can be caused by inherited muscle disorders, such as muscular dystrophies and metabolic myopathies [8,9].

## Case presentation

**Figure 1:**
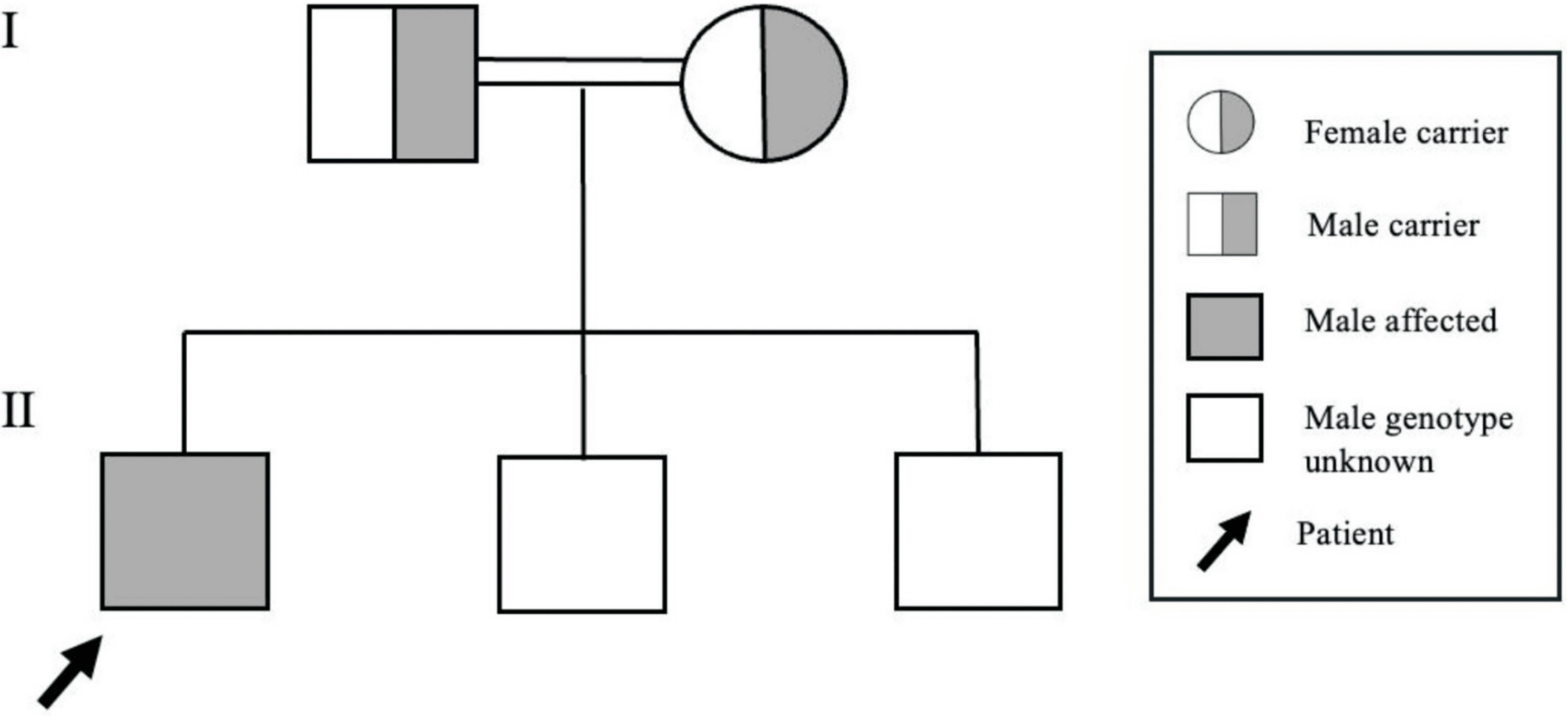
Pedigree of the patient's family demonstrating autosomal recessive inheritance of mucolipidosis type IV The proband (arrow) is a 10-year-old male with genetically confirmed mucolipidosis type IV. Both parents are heterozygous carriers of the pathogenic *MCOLN1 *variant. The patient has two younger brothers (ages seven and four) who are reportedly healthy but have not undergone genetic testing. Image created by the authors using PowerPoint (Microsoft® Corp., Redmond, WA)

A 10-year-old Bahraini boy, born at term after an uncomplicated vaginal delivery, was the first child of consanguineous parents. His birth weight was within the normal range. He developed neonatal jaundice lasting 10 days, which was treated with phototherapy, without a NICU admission. He has two healthy siblings. Family history is significant for glucose-6-phosphate dehydrogenase (G6PD) deficiency and sickle cell trait. There is no other family history of genetic or neurological disease. A pedigree analysis was constructed based on available family history and genetic testing results, demonstrating an autosomal recessive inheritance with both parents identified as carriers of the pathogenic *MCOLN1 *variant (Figure 1).

**Figure 2:**
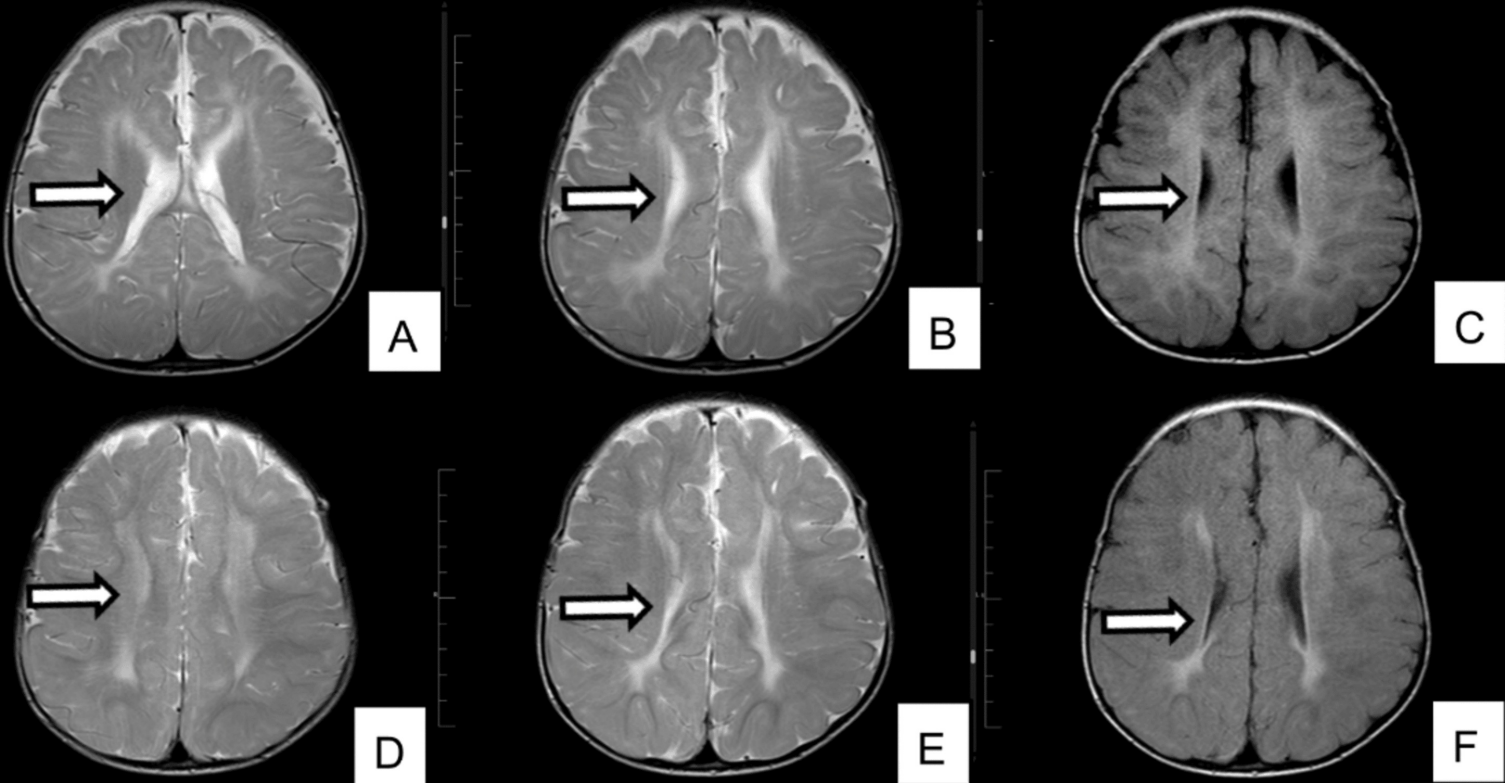
Brain MRI of a patient with mucolipidosis type IV obtained at the age of 15 months (A-C) and repeated at the age of two years (D-F) (A) Adjacent axial T2-weighted image confirming persistent periventricular T2 hyperintensity (arrow) and reduced white matter volume without cystic degeneration or basal ganglia signal abnormality. (B) Axial T2-weighted image demonstrating bilateral symmetric hyperintense signal within the periventricular white matter (arrow), most prominent along the anterior and posterior limbs of the internal capsule. Diffuse thinning of the corpus callosum is evident. (C) Axial T2-weighted fluid-attenuated inversion recovery (FLAIR) image demonstrating bilateral symmetric periventricular white-matter hyperintensity greatest along the anterior and posterior limbs of the internal capsule (arrow), with diffuse thinning of the corpus callosum; ventricles normal. (D) Adjacent axial T2-weighted image showing continued diffuse thinning of the corpus callosum (arrow) without hydrocephalus or cystic periventricular change. (E) Axial T2-weighted image demonstrating bilateral symmetric periventricular hyperintensities (arrow) that appear more prominent, consistent with delayed myelination/hypomyelination. (F) Axial T2-weighted FLAIR image showing persistent bilateral periventricular white-matter hyperintensity (arrow) and reduced white matter volume without cystic change; corpus callosum thinning persists.

From infancy, global developmental delay was evident, accompanied by marked axial hypotonia. He achieved sitting but never crawled or walked. Fine motor skills were limited to grasping objects, and speech remained restricted to a few single words. He had preserved social responsiveness. At 15 months of age, an MRI of the brain revealed delayed myelination, thinning of the corpus callosum, and non-cystic periventricular leukomalacia (Figures 2A-2C); repeat imaging at two years of age showed progression of these changes (Figures 2D-2F).

**Figure 3:**
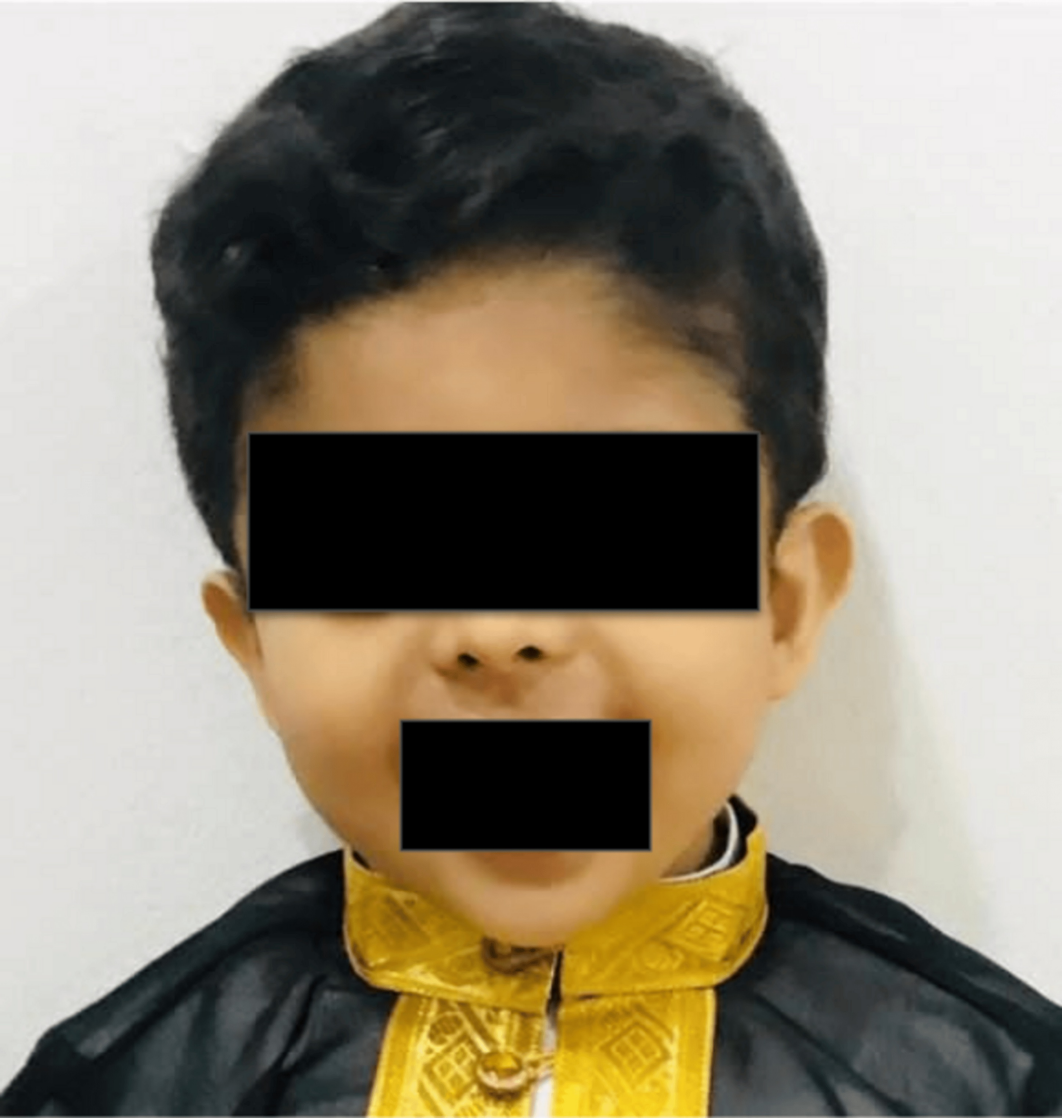
A child with mucolipidosis type IV Clinical photograph demonstrating subtle dysmorphic features, including epicanthal folds and midface hypoplasia.

The EEG performed for frequent extensor posturing during sleep-wake transitions was normal. Ophthalmological evaluation under anesthesia at age two years demonstrated corneal clouding and retinal degeneration, which progressed to severe visual impairment by school age. On clinical examination, epicanthal folds and midface hypoplasia were observed (Figure 3); however, these features are not clearly depicted in order to preserve patient identity.

Between the ages of one and four, the patient required several hospital admissions for gastrointestinal illness and dehydration. At 17 months, he presented with prolonged diarrhea and fever; ultrasound demonstrated colitis with mesenteric lymphadenopathy. He experienced recurrent gastroenteritis episodes in later years. He has had persistent anemia from combined G6PD-related hemolysis, iron deficiency, and poor nutrition, with hemoglobin often falling to 6.5-9 g/dL. He received iron supplementation along with occasional transfusions, the most recent at 10 years of age, when his hemoglobin level was 6.8 g/dL. The patient's detailed laboratory findings are presented in Table 1. 

**Table 1:** Laboratory results of a patient with mucolipidosis type IV WBCs, White blood cells; RBCs, Red blood cells; MCV, Mean cell volume; MCH, Mean cell hemoglobin; NR, No record

Laboratory test	Normal range	Patient age (years)								
		0.6	4.8	7.2	7.7	8.0	8.1	9.1	9.7	10.0
WBCs (L)	3.6-9.6×10^9^	NR	3.11	7.4	11.61	4.05	14.92	5.06	11.2	5.26
RBCs (L)	3.9-5.2×10^12^	NR	5.12	6.05	5.87	5.55	5.63	5.45	5.39	4.97
Hemoglobin (g/dL)	12.0-14.5	11.9	6.5	8.1	9	8.8	8.7	7.4	8.1	6.8
Hematocrit (%)	33-45	NR	27.3	28.5	30.7	29.5	29.5	29.9	30.3	26
MCV (fL)	80.0-97.0	NR	53.5	47.1	52.3	53.1	52.4	54.9	56.2	52.3
MCH (pg)	27.0-33.0	NR	12.6	13.4	15.3	15.9	15.5	13.6	15	13.7
Platelet (L)	150-400×10^9^	NR	418	375	418	379	NR	311	432	442
Creatine Kinase (U/L)	46-171	NR	NR	NR	NR	7887	7103	NR	NR	NR
Ferritin (µg/L)	16.0-323.0	NR	NR	NR	NR	NR	NR	NR	5.9	NR
Iron (μmol/L)	11.6-31.3	NR	4	NR	NR	NR	NR	NR	NR	2.7
Transferrin (g/dL)	2.15-3.65	NR	NR	NR	NR	NR	NR	NR	NR	3.66
Transferrin Saturation (%)	15-33	NR	NR	NR	NR	NR	NR	NR	NR	3

**Figure 4:**
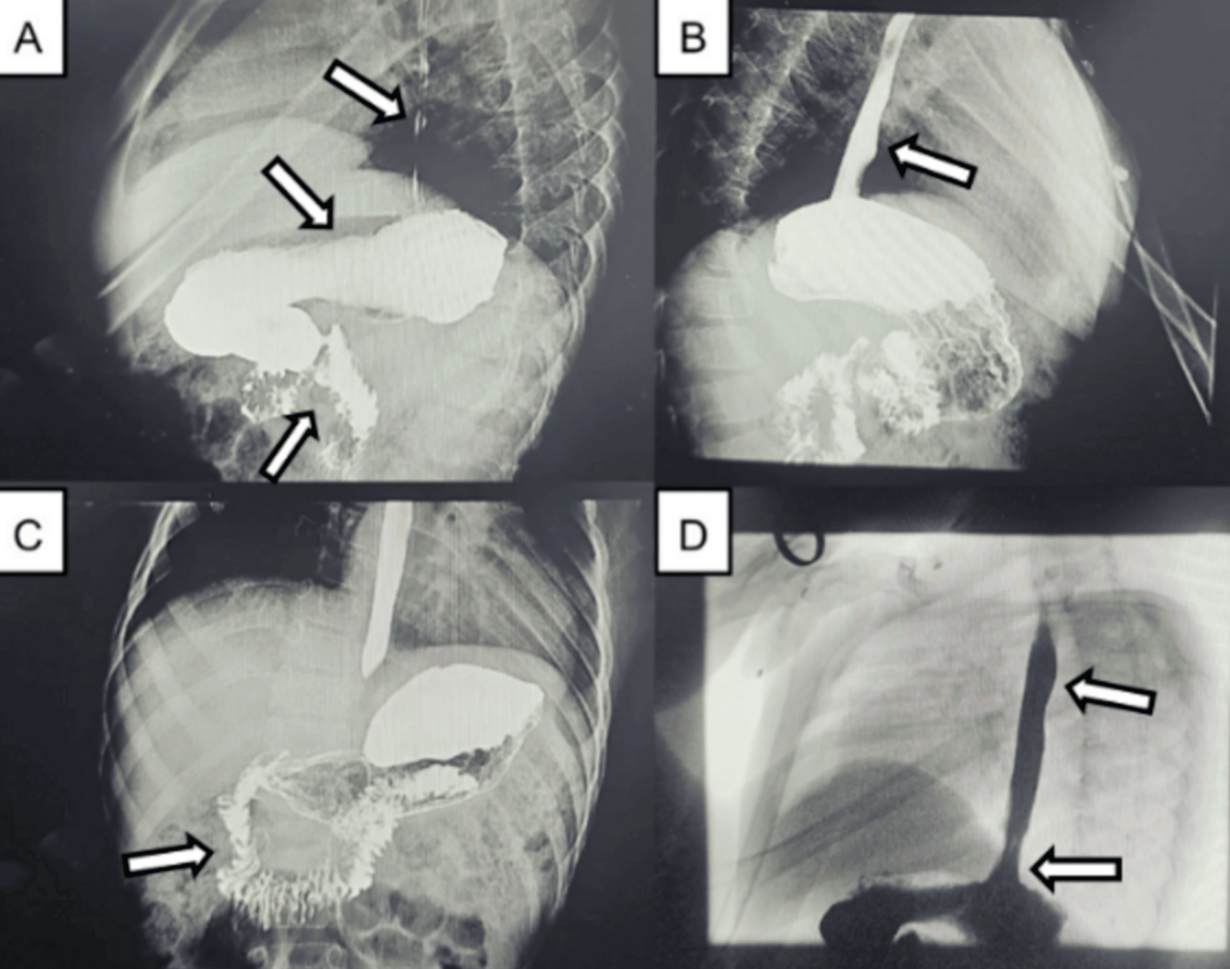
Barium swallow report of a patient with mucolipidosis type IV (A-C) Normal passage of contrast through the esophagus, stomach, and small bowel without structural obstruction. (D) Reflux of contrast into the esophagus during the water siphonage test, consistent with severe gastroesophageal reflux.

At age six, whole-exome sequencing confirmed a homozygous *MCOLN1 *variant (c.1336G>A; p.Val446Met), consistent with autosomal recessive MLIV. In this patient, the diagnosis was established on the basis of the characteristic clinical phenotype together with confirmatory molecular genetic testing. Genetic counseling was provided. From age seven onwards, feeding difficulties and gastroesophageal reflux became more prominent. At eight years of age, a swallow assessment and barium study demonstrated severe gastroesophageal reflux (Figure 4). Gastrostomy was recommended, but declined by the family. 

**Figure 5:**
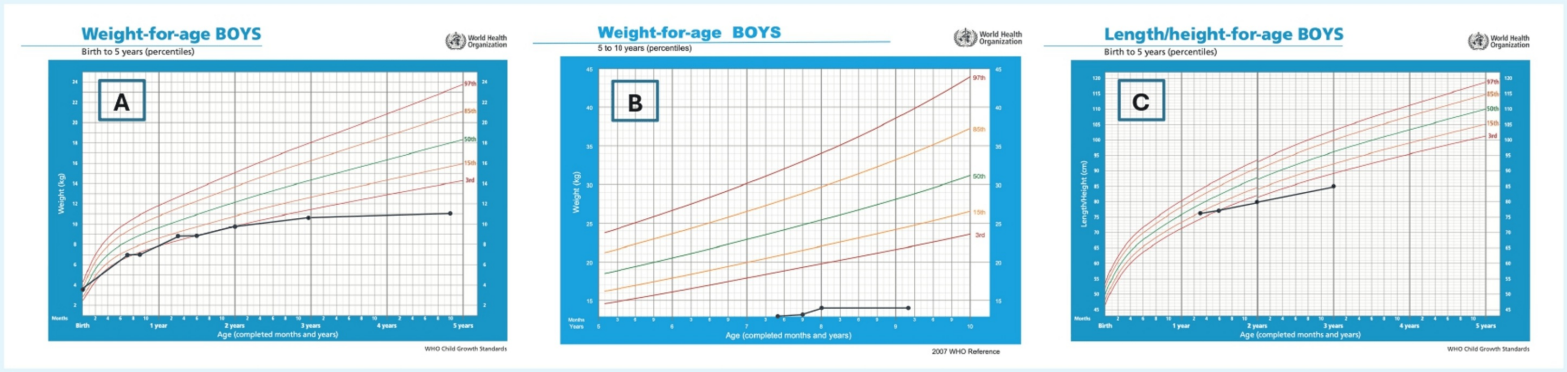
World Health Organization growth charts showing the anthropometric parameters of a child with mucolipidosis type IV (A) and (B) Weight-for-age measurements from birth to five years and five to 10 years, respectively. (C) Length/height-for-age measurements from birth to five years. Image created by the authors using PowerPoint (Microsoft® Corp., Redmond, WA)

By age 10, his weight was 15 kg, consistent with moderate malnutrition despite high-calorie supplementation (Figure 5).

An unusual feature in this case has been persistent hyperCKemia. At eight years of age, he presented with bilateral leg pain and inability to sit, initially assessed as a self-limited inflammatory musculoskeletal episode. Investigations revealed a CK of 7,887 U/L, falling to 1,927 U/L over two days (Table 2), with elevated lactate dehydrogenase (LDH), neutrophilia, and thrombocytosis.

**Table 2:** Serial serum creatine kinase (CK) measurements in a patient with mucolipidosis type IV Values are presented chronologically according to age at the time of testing.

Age (years, months, days)	Age-adjusted CK reference range (U/L)	Patient CK level (U/L)
1 year 3 months 16 days	≤228	372
8 years 0 months 8 days	27-62	7,887
8 years 0 months 10 days	27-62	1,927
8 years 2 months 28 days	27-62	3,302
8 years 2 months 29 days	27-62	2,537
8 years 3 months 0 days	27-62	7,103
10 years 0 months 8 days	46-171	1,216
10 years 0 months 9 days	46-171	1,286

**Figure 6:**
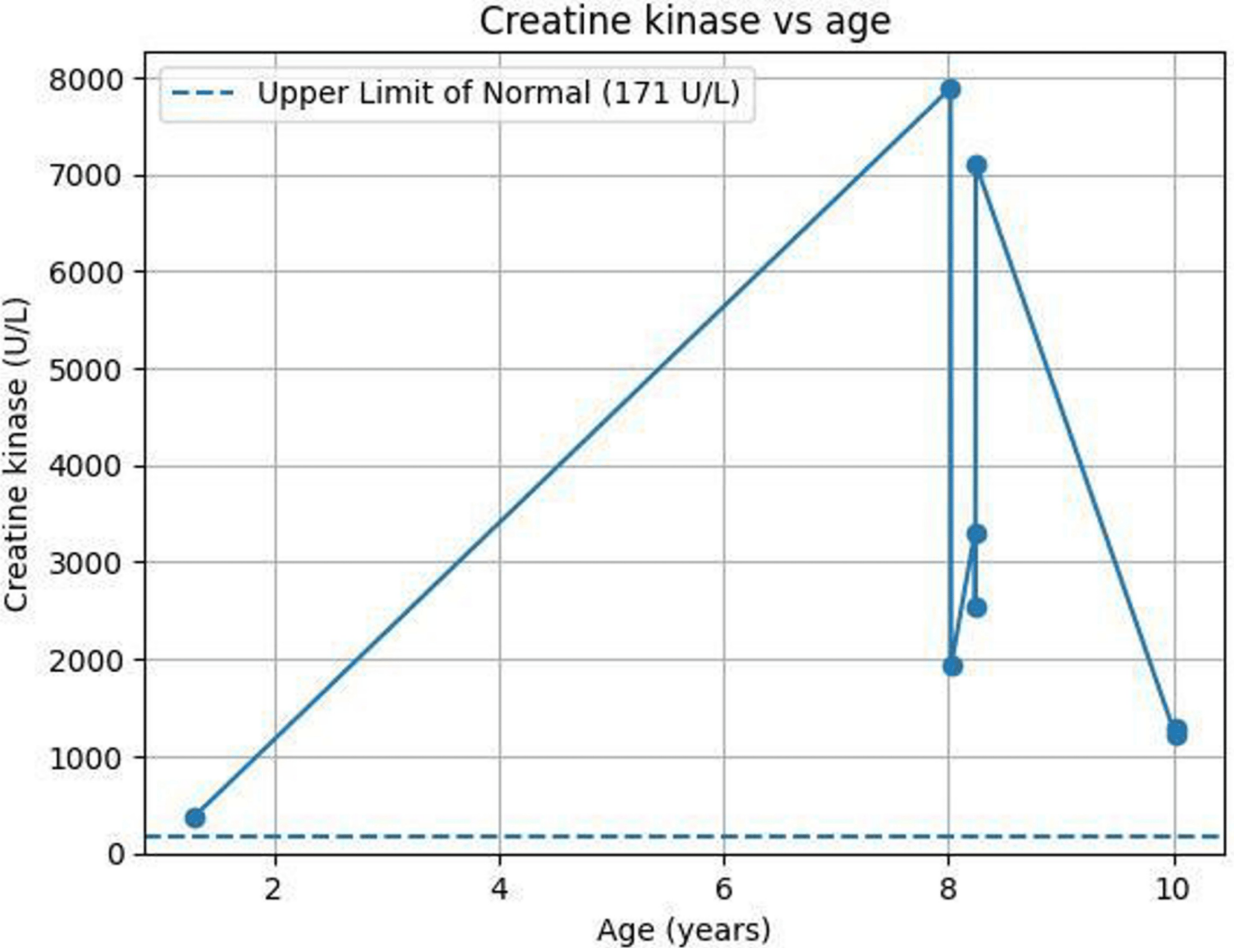
Longitudinal serum creatine kinase levels in a patient with mucolipidosis type IV Serial CK measurements demonstrate persistent hyperCKemia with marked transient elevations at approximately eight years of age. The dashed line indicates the upper limit of normal (~171 U/L). Peak elevations were temporally associated with intercurrent viral illnesses and subsequently declined, although CK levels remained persistently above normal.

The patient was diagnosed with influenza B and treated for myositis with naproxen and supportive care. CK has remained intermittently raised on later admissions, an atypical finding in MLIV and suggestive of associated myopathic involvement (Figure 6). 

**Figure 7:**
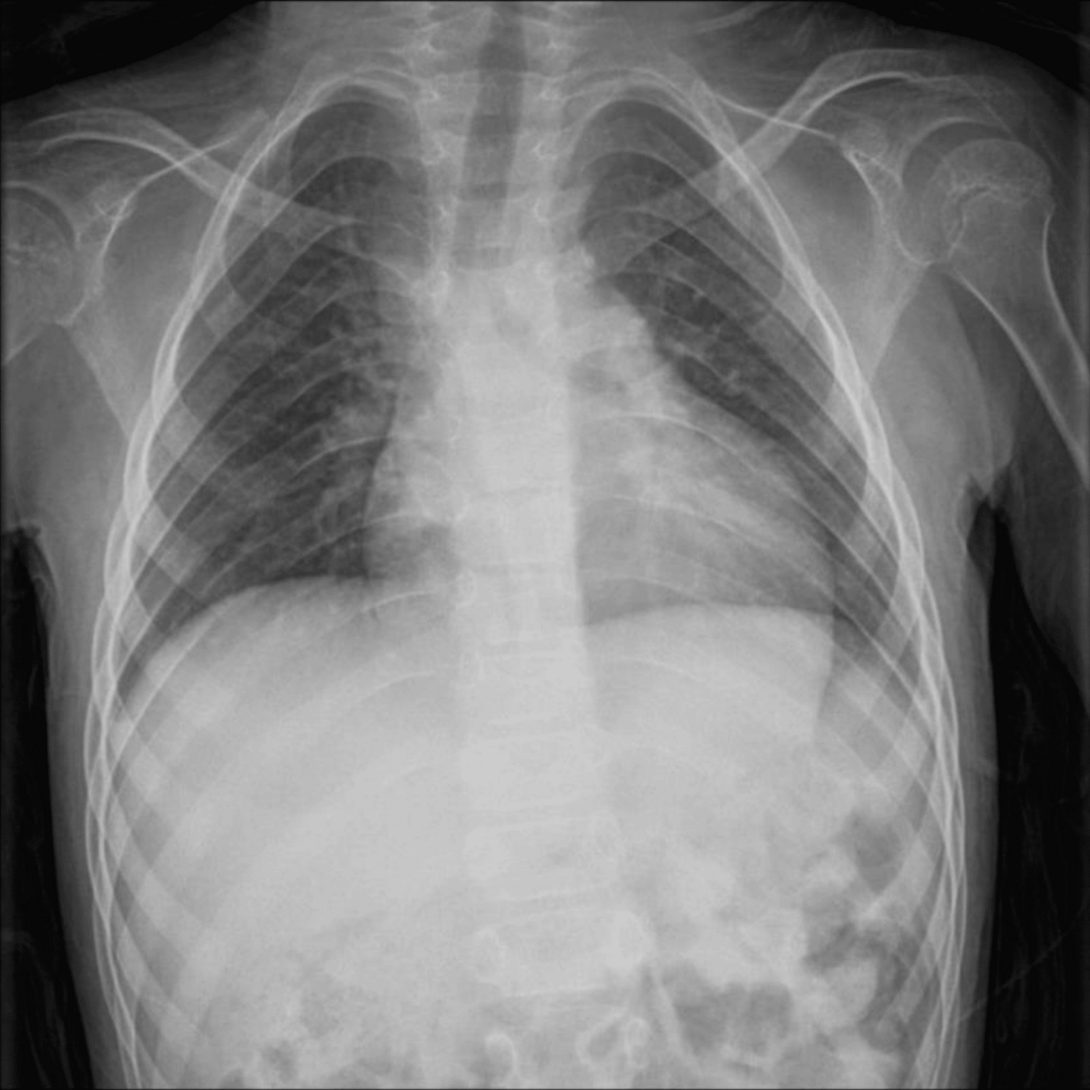
An antero-posterior chest radiograph of a nine-year-old patient with mucolipidosis type IV Chest radiograph demonstrating an increased cardiothoracic ratio (~58%), raising suspicion for cardiomegaly.

A chest radiograph at age nine demonstrated an increased cardiothoracic ratio (~58%), raising suspicion of cardiomegaly. Echocardiography was recommended/referred for, but is pending at the time of writing. Detailed radiology findings are presented in Figure 7. 

Currently, at age 10, he is non-verbal, severely visually impaired, and fully dependent for activities of daily living. He can sit with support but cannot walk. Neurologically, he has spastic quadriparesis with brisk reflexes and early contractures. He has recurrent respiratory infections but no documented seizures. He remains under multidisciplinary follow-up in neurology, genetics, gastroenterology, ophthalmology, dietetics, and physiotherapy. Despite his severe disabilities, he maintains social awareness and responds to caregivers, and his family remains closely involved in his care.

## Discussion

MLIV is a rare autosomal recessive disorder with an estimated incidence of approximately one in 40,000 live births, and the majority of reported cases occur in individuals of Ashkenazi Jewish ancestry due to founder variants in *MCOLN1 *[7]. Approximately 100-120 patients have been described worldwide [10]. In Ashkenazi Jewish populations, carrier frequencies approach 1%, reflecting the impact of well-characterized founder mutations and structured community screening programs [7,11].

Outside these founder populations, MLIV remains infrequently reported and is likely underrecognized. In Saudi Arabia, a 2013 case series described five affected children from a consanguineous family carrying a novel *MCOLN1 *mutation (p.Y436C) [11]. Similarly, six patients from two unrelated Omani families were found to harbor a novel splice-site mutation (c.237+5G>A) [4], and four siblings from Iran were reported with a novel homozygous variant (p.T121M) associated with severe neurologic manifestations [10]. In Turkey, an affected child of consanguineous parents was found to carry a homozygous *MCOLN1 *c.1367C>T (p.S456L) mutation, inherited heterozygously from each parent. The patient showed the typical MLIV phenotype, along with unique findings, such as micrognathia, fifth finger clinodactyly, and posterior internal capsule involvement on MRI [12]. Despite these reports, population-level prevalence data across most Middle Eastern countries remain unavailable. The absence of systematic screening and rare-disease registries in many Arab populations suggests that additional cases may remain undiagnosed. In this context, the present case represents an important contribution to the limited regional literature.

To our knowledge, no published case reports or epidemiological studies have documented MLIV in Bahrain; several neighboring Gulf states, such as Iraq, the United Arab Emirates, Kuwait, and Qatar; or Middle Eastern countries, including Egypt, Jordan, Lebanon, Syria, and Yemen. In contrast to the limited family series reported from other Middle Eastern countries, this case represents the first genetically confirmed report of MLIV from Bahrain. Whether the absence of prior reports reflects true rarity or underdiagnosis remains uncertain; however, limited access to rare-disease registries and comprehensive molecular testing in many Arab populations likely contributes to under-recognition. In this context, our case expands the geographic and genetic spectrum of MLIV and highlights the need for broader surveillance and molecular characterization in underrepresented populations.

A wide spectrum of *MCOLN1 *pathogenic variants has been described in MLIV. The most prevalent mutation in Ashkenazi Jewish populations is a splice-site mutation in intron 3, accounting for approximately 72% of Ashkenazi MLIV alleles and resulting in defective splicing with absent functional mucolipin-1 [13,14]. A 6.4-kb partial gene deletion encompassing exons 1-7 represents a second founder mutation, present in approximately 23% of Ashkenazi alleles, and similarly leads to severe loss of protein function. Beyond these founder variants, additional pathogenic mutations include nonsense and frameshift variants, as well as missense substitutions affecting conserved regions of the gene outside the transmembrane domains, lipase motif, and nuclear localization signal. An in-frame deletion (F407del), identified in compound heterozygosity with the intron 3 splice-site mutation, has been associated with a comparatively milder phenotype [13,14]. In our patient, genetic testing identified the *MCOLN1 *c.1336G>A (p.Val446Met) variant, which represents a missense substitution and is therefore mechanistically distinct from the common Ashkenazi founder splice-site and deletion mutations discussed above. As a missense variant, it is predicted to alter TRPML1 protein structure or function rather than abolish protein production entirely, which may contribute to phenotypic variability [13-15]. This missense mutation has been reported previously in clinical sequencing studies of neurodevelopmental disorders associated with MLIV [16].

Genotype-phenotype correlations in MLIV suggest that severe neurologic impairment is most frequently observed in patients harboring the major Ashkenazi splice-site mutation and/or the 6.4-kb deletion, both of which result in markedly reduced or absent *MCOLN1 *mRNA expression and loss of mucolipin function [13,14]. These patients typically exhibit early motor delay, corpus callosum dysgenesis, and profound visual impairment. In contrast, mutations that preserve partial mucolipin activity are associated with milder neurologic outcomes. For example, compound heterozygosity for the major splice-site mutation and the F407del variant has been linked to improved motor development and near-normal corpus callosum morphology. Similarly, variants associated with preserved mRNA levels may permit limited independent gait and better gross motor function, even in the presence of callosal abnormalities [15]. Consistent with these findings, low or absent *MCOLN1 *expression correlates with more severe neurologic outcomes, whereas preserved expression is associated with improved motor function and larger corpus callosum area [13,14].

Further refinement of prognostic expectations can be derived from the topological clustering of missense variants. Mutations located within the loop between TM1 and TM2 are generally associated with milder phenotypes. Variants affecting TM3 may result in relatively mild neurologic impairment, although progressive retinal involvement can occur. TM4-associated variants have been described in some of the mildest phenotypes, whereas substitutions between TM5 and TM6 - corresponding to the channel pore region - are typically linked to more severe disease. Notably, a c.1256G>C variant in exon 11 (TM4-TM5 loop) has been associated with a severe phenotype [15].

Clinically, MLIV is characterized by early-onset global developmental delay and hypotonia, with developmental concerns typically emerging between 6 and 15 months of age [1,10,11,13,17]. Most affected children demonstrate delayed motor milestones and severely limited expressive language, frequently progressing from hypotonia to spastic quadriparesis during adolescence [5]. This trajectory was clearly observed in our patient, who achieved independent sitting but never attained crawling or ambulation, and whose expressive language remained restricted to single words. Over time, spasticity developed and was clinically interpreted as cerebral palsy, reflecting the characteristic neuromotor evolution of MLIV.

Progressive ophthalmologic degeneration represents a hallmark feature of MLIV, typically beginning with corneal clouding and advancing to retinal degeneration, with severe visual impairment often evident by early adolescence [5,17,18]. Our patient followed this expected course, developing progressive visual decline over time. Additional manifestations, such as nystagmus, photophobia, and strabismus, have been reported along with ptosis, optic nerve atrophy, and intermittent corneal erosions [1,10,13,17,18].

Feeding difficulties due to oromotor dysfunction are also common [5,7], and our patient experienced early feeding challenges with subsequent dysphagia and severe gastroesophageal reflux confirmed on swallow assessment. Although coarse facial features are generally absent, subtle dysmorphic findings may be present [4,10,17]. In our patient, epicanthal folds and midface hypoplasia were observed. Further, these features are consistent with the classical systemic profile of MLIV; hepatosplenomegaly and major skeletal abnormalities were not present on examination [5,17,18].

Beyond the core phenotype, our patient exhibited additional clinical findings, including neonatal jaundice, iron deficiency anemia, and anemia secondary to G6PD deficiency. The presence of sickle cell trait represents a coincidental genetic comorbidity rather than a direct manifestation of MLIV. Episodes of extensor posturing during sleep-wake transitions were evaluated, with no confirmed seizure disorder. Neuroimaging demonstrated periventricular leukomalacia, a finding not considered diagnostic for MLIV but potentially reflective of disease severity in cases with significant motor impairment [5]. Moderate malnutrition and severe gastroesophageal reflux further compounded feeding and growth challenges. Parental consanguinity is consistent with the autosomal recessive inheritance pattern of MLIV and likely contributed to disease manifestation in this patient [4].

MLIV is increasingly recognized as a slowly progressive neurodegenerative disorder rather than a static encephalopathy. Affected individuals often experience gradual worsening of motor deficits during childhood and adolescence, with limited independent ambulation and persistent language impairment [1,19]. Although overt seizures are uncommon, epileptiform EEG abnormalities have been described [1]. In addition to neurologic involvement, gastrointestinal manifestations - particularly achlorhydria with secondary iron deficiency - are highly prevalent [1,4,5]. Chronic dysmotility and constipation may further complicate management. Progressive renal impairment has also been reported in adulthood. Despite significant morbidity, many individuals survive into adulthood, although life expectancy may be reduced due to complications of severe neurologic disability [1].

A distinctive aspect of this case was the persistent elevation of serum CK across multiple time points. Mild elevation was first documented at one year and three months of age (372 U/L), followed by sustained hyperCKemia ranging from 1,200-3,200 U/L, with intermittent peaks exceeding 7,000 U/L (7,103 U/L and 7,887 U/L) during symptomatic influenza B and bronchitis episodes at eight years of age (Table 2). More recent measurements at 10 years of age remained mildly to moderately elevated (1,216-1,286 U/L). Although elevated CK is not considered a classical biochemical hallmark of MLIV, rare reports have documented presentations with congenital myopathy and markedly elevated CK levels. Zambon et al. described persistent hyperCKemia up to 15,000 U/L in association with lysosomal storage myopathy attributable to *MCOLN1 *dysfunction. These findings suggest that CK elevation in MLIV may reflect secondary myopathic involvement related to impaired lysosomal function and defective sarcolemmal repair [1,5]. Importantly, no established correlation exists between CK levels and genotype, phenotypic severity, or prognosis in MLIV [1]. Recognition of this association is clinically relevant, as unexplained hyperCKemia may initially prompt investigation for primary muscular dystrophies, potentially delaying diagnosis of MLIV in patients with compatible neuro-ophthalmologic features [5]. The longitudinal CK profile observed in our patient therefore broadens the recognized biochemical spectrum of MLIV, particularly within underrepresented Middle Eastern populations [1,5,14].

## Conclusions

MLIV is a rare autosomal recessive lysosomal disorder with a characteristic neuro-ophthalmologic and gastrointestinal phenotype but variable systemic expression. This report represents the first genetically confirmed case of MLIV from Bahrain, expanding the geographic spectrum of the disease in the Middle East. Notably, the persistent and intermittently marked elevation of CK observed in this patient suggests that secondary myopathic involvement may be an underrecognized component of the MLIV phenotype. Recognition of this association is clinically important, as unexplained hyperCKemia may prompt evaluation for primary muscular dystrophies and delay consideration of MLIV in appropriate clinical contexts. Broader molecular surveillance and increased awareness in underrepresented populations are essential to improve early diagnosis and refine understanding of the disease spectrum.
